# Science students' perspectives on how to decrease the stigma of failure

**DOI:** 10.1002/2211-5463.13345

**Published:** 2021-12-13

**Authors:** Krystal Nunes, Sherry Du, Riya Philip, Mohammed Majd Mourad, Zainab Mansoor, Nicole Laliberté, Fiona Rawle

**Affiliations:** ^1^ Department of Biology University of Toronto Mississauga Canada

**Keywords:** failure in STEM, productive failure, resilience, stigma of failure, student engagement

## Abstract

Failure is hard‐wired into the scientific method and yet teaching students to productively engage with failure is not foundational in most biology curricula. To train successful scientists, it is imperative that we teach undergraduate science students to be less fearful of failure and to instead positively accept it as a productive part of the scientific process. In this article, we focus on student perceptions of the stigma of failure and their associated concerns to explore how failure could be better supported within and beyond a university context. Through a survey of first‐year biology students, we found that societal and familial pressures to succeed were the greatest contributing factors to students' fear of failure. In student suggestions on how to reduce the stigma of failure within and beyond the university context, the most common theme identified across both contexts was for increased discussion and open communication about experiences of failure. Importantly, student comments in this study bring attention to the role of factors beyond the classroom in shaping student experiences of failure within their biology courses.

## Graphical abstract

To train successful scientists, we must teach undergraduate students to be less fearful of failure as it is a productive part of the scientific process. An investigation of student perceptions of failure found that societal and familial pressures were the greatest contributing factors to fear of failure. To decrease the stigma of failure, students suggest increased discussion of failure experiences.

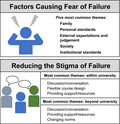

**Abbreviations**
ANOVA: analysis of variance
STEM: science, technology, engineering, mathematics

Perseverance through setbacks and failures is an important trait of successful scientists as learning from failure is hard‐wired into the scientific method [1, 2, 3]. Apart from accidental discoveries, failed experiments can lead to refinement and improvement of the experimental design, revision of the original hypothesis, and redirection of research goals. Failure can inspire a shift in viewpoint, leading to new questions and creative solutions to scientific problems [4]. Therefore, to support greater success in future science‐based careers, undergraduate science education should teach students to learn from and embrace failure. However, teaching students to engage in risk‐taking behaviors is challenging for instructors as failure can be associated with negative stigma and risk aversion [5].

Successful scientific careers are often marked with more failures than successes, yet failure is rarely explicitly taught in undergraduate, science, technology, engineering, mathematics (STEM) classrooms [2]. For example, students are often presented with highly detailed laboratory instructions that have been carefully designed and tested to produce a desired outcome [1, 3]. While such experiences are valuable in teaching lab techniques, they do not accurately represent the complete scientific method as revision, refinement, and troubleshooting are critical parts of this cyclical process. Similarly, the content of undergraduate science textbooks is typically filled with stories of historically successful discoveries and innovations. To stress the importance of failure within the scientific process, instructors could include narratives of failure in their course material. However, learning to recover from, and reflect on, setbacks is an emotional process that requires guidance and support. Discussions of failure in an abstract manner, as opposed to personally experiencing failure and engaging with the stressor, may be insufficient for students to develop coping skills and resilience [6]. To best prepare STEM students to be successful, resilient scientists, we argue it is necessary to provide opportunities to productively engage with failure.

The ability to experience and persevere through challenges is an important part of the learning process [1, 2, 7]. Failures provide opportunities to reflect on and modify study habits, and assessments specifically designed for productive failure can increase understanding of concepts [2, 8]. For instance, failure following a difficult, low‐weight assessment can improve students’ understanding of learning expectations, identify key issues with their work or study habits, and encourage them to seek additional support [9]. Overall, failure can play an integral role in the learning process as it can improve resilience, allow for reflection and improved study habits, and increase information retention and academic performance [8, 10, 11, 12].

Despite the benefits to the learning process, the term ‘failure’ often carries negative connotations in both academic and non‐academic settings, which may contribute to fear of failure and risk avoidance. Although the definition of failure will depend on one’s personal goals, values, and expectations, research suggests that most early undergraduate STEM students do not view failure as a positive learning experience [1, 2]. Negative perceptions of failure can evoke emotions of anxiety, low self‐confidence, and reduced perseverance, and a fear of failure can create an aversion to future risk taking and engagement with challenges [2, 5, 13]. The ability of learners to effectively develop resiliency is highly context dependent and will be influenced by their individual expectations, past experiences, the structure of the educational system, and their interactions with those within and outside the institution [14]. Some of these factors may contribute to the negative stigma associated with failure, consequently interfering with one’s development of resiliency. Furthermore, these factors may be heightened in populations traditionally underrepresented in STEM fields such as women, ethnic minorities, and other marginalized groups [2, 15, 16, 17].

Fear of failure may lead to risk avoidance and maladapted coping techniques [18]. Resiliency and productive coping strategies are core transferrable skills, but much of the existing research on fostering the development of these skills in students has focused on growth mindsets and goal orientation [1, 19]. Although mindset and goal orientation are important to developing resiliency, there remains an emotional cost to developing these skills that may be heightened by the negative stigma surrounding failure. The purpose of this study is to examine undergraduate science student perspectives on how to decrease the stigma of failure both within and beyond the university context. We investigate contributing factors to one’s fear of failure, and explore student perspectives on minimizing the shame, fear, and stigma associated with failure.

## Methods

### Survey administration

The University of Toronto Mississauga is located in the diverse urban population of Mississauga, Ontario, Canada. All students enrolled in BIO152: Introduction to Evolution and Evolutionary Genetics during the Fall 2019 term were invited to participate in an online survey (*n* = 822). This introductory biology course is mandatory for those pursuing a biology‐related major/minor or specialist degree in the Life Sciences program. Most enrolled students were in their first year of undergraduate studies (96.3%), and the majority of students identified as female (71.2% female, 27.5% male, 1.3% undisclosed) based on university records. Participants that completed the anonymized survey received a course bonus point regardless of whether they consented to include their data in the research analyses. Survey completion was recorded in the grade sheet of the course management system, and responses were anonymized by a third‐party following submission.

A total of 574 students gave informed consent to have their survey responses included in data analyses (consent rate of 69.8%). Of consented respondents, 76.1% self‐identified as female; 21.7% as male; 0.5% as nonbinary; 0.2% as transgender; 0.3% of respondents stated that their gender was not listed; and 1% preferred not to answer.

### Survey questions

The survey consisted of questions on student perceptions of failure and stigma. Students were presented two Likert‐scaled questions. The first asked students to rate how strongly they felt five factors (family, friends, teachers, bosses/supervisors, and societal pressures) contributed to their fear of failure on a five‐point scale from ‘strongly agree’ to ‘strongly disagree’. Although both interpersonal and intrapersonal sources for fear of failure have been identified [13, 20], our Likert categories focused on interpersonal factors to broadly identify aspects of lived experience and social interactions that influence fear of failure. However, we complimented the Likert‐scaled questions with open‐ended responses on perceptions of failure, outlined below. The second Likert‐scaled question ask students to rate their openness to failure at the end of the course on a five‐point scale from ‘strongly not open to failure’ to ‘strongly open to failure’.

Students were presented three open‐ended questions: (a) In your opinion, what factors cause your fear of failure; (b) in your opinion, what specifically can be done to decrease the stigma of failure within the university context, and (c) in your opinion, what specifically can be done to decrease the stigma of failure beyond the university context? In a separate reflective assignment, BIO152 students were presented with the question, ‘What resources do you think students need to help them embrace and bounce back from failure?’ We have also included those anonymized responses in our analyses.

### Response rate

Students were not required to answer all the questions if they did not feel comfortable doing so. Of students who gave informed consent to participate in the survey, a response rate of 98.3% (*n* = 564) was achieved for four of the Likert‐scaled factors contributing to failure (family, friends, teachers, bosses/supervisors), while the response rate of the final factor (societal pressures) was 98.1% (*n* = 563). A response rate of 99.1% (*n* = 569) was achieved for the Likert‐scaled question on openness to failure. Following the exclusion of blank submissions, a response rate of 98.6% (*n* = 566) was achieved for the open‐ended question on factors causing one’s fear of failure, and a response rate of 95.8% (*n* = 550 responses) was achieved for the open‐ended questions on decreasing the stigma of failure within and beyond the university context. The open‐ended question of recommended resources to improve resilience originated from a separate BIO152 reflective assessment; *n* = 880 suggestions were provided.

### Data analysis

Data were analyzed using Creswell’s coding protocol [21]. Themes and subthemes were identified in the open‐ended responses; two coders processed an equal number of survey responses. Some responses were assigned multiple codes if they aligned with more than one theme/subtheme. Themes and subthemes underwent validation to ensure consistency in application of the coding scheme.

Descriptive Likert‐scaled responses were converted to a numerical scale for analysis (for factors contributing to fear of failure, 1 = strongly disagree, 2 = disagree, 3 = neutral, 4 = agree, 5 = strongly agree; for openness to failure, 1 = strongly not open to failure, 2 = not open to failure, 3 = neutral, 4 = open to failure, 5 = strongly open to failure). The importance of the five factors (friends, bosses/supervisors, teachers, family, and societal pressures) were compared using a one‐way ANOVA and Tukey *post hoc* test. Although Likert data are ordinal, parametric tests have been found to be robust to violations of normal distribution assumptions and inequal variance, particularly when used with large sample sizes of Likert data [22, 23]. A Kruskal–Wallis test was used to determine whether a relationship existed between the Likert‐scaled question on openness to failure and coded themes to the question ‘In your opinion, what factors cause your fear of failure?’ Statistical analyses were performed in r, ver. 3.6.1 (R Core Team 2019, Vienna, Austria).

### Ethics

This research was approved by the University of Mississauga Delegated Ethics Review Committee in Fall 2019.

## Results

### Factors contributing to students' fear of failure

**Fig. 1:**
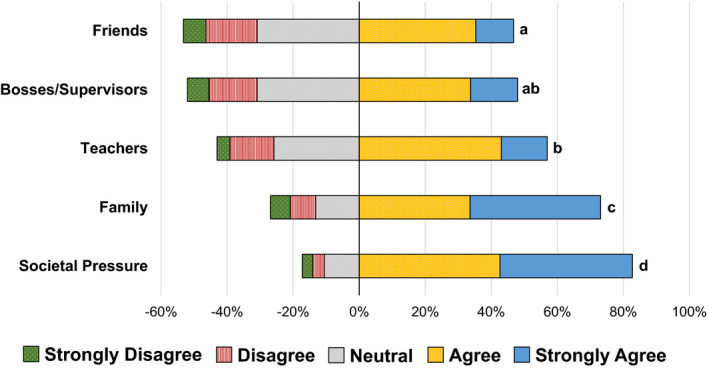
BIO152 students at the University of Toronto Mississauga used a five‐point Likert scale to answer the question ‘Did the following contribute to your fear of failure?’ based on five provided factors. Factors sharing the same letter do not significantly differ based on a one‐way ANOVA and Tukey’s *post hoc* test (F(4, *n* = 574) = 69.1, *P* < 0.0001).

‘Societal pressures’ had the greatest average Likert score as a factor contributing to students’ fear of failure (x̄ = 4.12, SD = 0.96; *F*(4, *n* = 574) = 69.1, *P* < 0.0001), followed by ‘family’ (x̄ = 3.93, SD = 1.17), ‘teachers’ (x̄ = 3.50, SD = 1.01), ‘bosses/supervisors’ (x̄ = 3.35, SD = 1.09), and lastly ‘friends’ (x̄ = 3.29, SD = 1.07; Figure 1). 82% of respondents agreed or strongly agreed that societal pressures contributed to their fear of failure. For example, one student stated, ‘I think people need to accept failure and not look down upon someone who has failed … because what happens is that you are afraid to share your failure in fear of people judging and talking about you’. 73% of respondents agreed or strongly agreed that family contributes to their fear of failure, with most responses identifying parent–student relationships. One student wrote, ‘The fact that my family immigrated from the other side of the world and my parents sacrificed a ton for my siblings and I to have all the opportunities we wanted. It’s a fear of disappointing them or making them think coming here wasn’t worth it…I don’t want to be the family failure'.

‘Bosses/supervisors’ and ‘friends’ scored similarly; 48% of respondents agreed or strongly agreed that bosses/supervisors contributed to their fear of failure, and 47% agreed or strongly agreed to friends as a contributing factor. ‘Bosses/supervisors’ and ‘friends’ also had the greatest proportion of respondents identifying them as neutral contributors to their fear of failure (both had 31% of respondents identify them as neutral) compared to the remaining three factors. 57% agreed or strongly agreed that teachers were a contributing factor to their fear of failure.

Although there was overlap in themes provided in the Likert question (society, family, peers, teachers), responses to the open‐ended question ‘In your opinion, what factors cause your fear of failure?’ resulted in unique themes emerging (Table 1). Seven new themes were identified: personal standards (23%), external expectations and judgment (18%), school/institutional standards (12%), consequences/permanent setback (9%), self‐worth (6%), past experiences (4%), and financial concerns (3%). However, the most common factor in the open‐ended responses was family (37%). Analysis of factors causing fear of failure and students’ openness to failure found no significant relationship (Kruskal–Wallis chi‐squared = 15.621, df = 13, *P*‐value = 0.2702).

**Table 1:** Coded themes for student responses to the open‐ended question, ‘In your opinion, where does your fear of failure come from?’ (*n* = 565).

Theme (includes percentage of responses coded to each theme^a^)	Description of Theme	Example Response
Family (37%)	Familial expectations of performance. References parents, siblings, and/or extended family.	‘I think my fear of failure mostly come from my parents and how they want me to succeed in school. If I receive a failing grade, I am more concerned about how they would feel about it rather than how I actually feel about it’.
Personal Standards (23%)	The goals of the student and the expectations they place on themselves. References to self‐motivation and desire to succeed.	‘My fears of failure come from mostly myself and the pressure I place on myself in order to do well. I know how much my parents have done for me in order to attend university. I feel that the least I can do is make them proud by succeeding and doing well in all of my classes…’
External Expectations and Judgment (18%)	Describes a pressure to perform and succeed based on the expectations of others, although no specific individuals/groups are mentioned. Often identifies a fear of being judged or criticized.	‘… People often tend to judge others for not meeting certain standards, or doing things the "right way" (aka. their way). When this happens, the person is criticized and made to feel as if they are not good enough. The fear here is having people talk and laugh about my failures and using the failures to create their own opinion of who I am as a person. In short, my failures do not determine who I am as a person, my character and personality. I am afraid of being seen as ‘lesser‐than’ by other people due to my failures’.
Society (16%)	Identifies the negative stigma associated with failure and/or cultural expectations. May describe concerns of failure impacting the position one holds in their community.	‘In my opinion, the fear of failure originated from social standards and academic requirements. Those who avoid failure fear that they will be unable to attain social and academic requirements necessary to succeed in life. Therefore, our society [does] not associate success with failure…’
School/Institutional Standards (12%)	Describes the consequences of failure that are a part of the educational system, such as the inability to earn a course credit, being placed on academic probation, etc.	‘… Its easy to preach about the importance of failure and list a few ways it has helped, but it doesn't change the fact that every little thing you do in school is graded and people are passed of failed, which can ruin someone chance at getting into a program or school. This is what I think about when I think about failing something which greatly contributes to my fear’.
Consequences/Permanent Setback (9%)	Fear that failure will lead to undue repercussions. Includes responses that describe failure as an event from which one cannot recover, leading to negative effects that will permanently impact one’s goals/future.	‘I am afraid of failure for a number of factors, but mainly I am afraid of what failure will do to me. I fear that I will never return to being myself again after I fail, I fear that my life will become miserable and sad, and lastly I fear that failure is irreversible. I also fear people's opinions, whether they will abandon me, or even worse be disappointed in me. In general, I am afraid of the unknown, and that is essentially what failure is, because I don't know what my life will look after I fail’
Peers (8%)	Includes reference to both friends and classmates. Describes self‐comparison to peers or judgment	‘My fear of failure comes from people who I am surrounded by. Specifically, my friends being more successful than me, academically, as it makes me feel like I am falling behind and am not smart enough to achieve good marks’.
Self‐worth (6%)	Connects failure to one’s personal value. Describes failures as a reflection of worth, personal image, self‐esteem, etc.	‘My fear of failure stems from not feeling good enough as a person and being scared to let others down. I often felt like a burden to others and I have been scared to upset others by failing’.
Past Experiences (4%)	Responses described either (a) negative experiences with past failures, leading to an aversion to risk taking, or (b) a lack of experience with failure, resulting in fear of the unknown	‘I feel like my fear of failure comes from inexperience. In my life, I haven't experienced any big failures so I lack exposure. Failing to me would be a new experience that I’m not comfortable with’.
Teachers (3%)	Includes interactions with elementary and high school teachers.	‘I think my fear of failure comes from high school…When I got to high school, specifically grade 11 and 12, teachers would always be like ‘if you can't do this then how will you do in [university],’ … ‘if you're one second late in handing something in they won't accept it’ and it goes on and on. Statements like these made me think that I can't afford to fail because if I fail in high school then imagine [university]. So failure was always seen as a ‘dumb person’ thing in high school and teachers would tell us [university] is even worse. So I definitely carried those thoughts with me to [university] even though I shouldn't have’.
Financial Concerns (3%)	Those who fear the monetary repercussions of failure, such as the cost of repeating a post‐secondary course.	‘I believe that my fear of failure comes from the fact that I am a first generation immigrant and my parents could not afford to go to school so they did not and I feel like I do not belong in academia. Also, I do not want to retake a course as it is expensive and I cannot afford to’.
Nondescript (3%)	Responses that did not answer the question.	N/A

^a^ Sum of response rates for each main theme does not equal 100% as some responses included multiple themes.

### Suggestions to reduce the stigma of failure within the university context

Eight themes were identified for addressing stigma within the university context. ‘Discussion/conversation’ was the most common theme (47%) with many responses emphasizing open discussion and sharing of failure experiences as indicated by the most common subtheme ‘Experiences’ (Table 2). As stated by one student, ‘In my opinion, I think everyone, especially in a university environment should openly talk about failure as if it is normal, including professors, students, peers, etc. I think many students fear it and avoid talking about failure because they feel as if they are being judged for it. Talking about it more and normalizing it will decrease the stigma around failure, making it easier for students to cope with it’.

**Table 2:** Coded themes for student perspectives on decreasing stigma of failure within the university context (*n* = 553).

Main Theme (includes percentage of responses coded to each theme^a^)	Subthemes (in order of rank)
Discussion/Conversation (47%) Inclusion of failure narratives. Increased communication on the topic of failure and resilience.	Experience: Sharing personal experiences or examples of failure and how the individual bounced back Positive approach: Promote welcoming conversations with less judgment/stigma toward failure Platform: Discuss failure through different avenues, including social media or anonymous comment submissions Teaching methods: Incorporation of more interactive class sessions rather than continuous content delivery Failure rate statistics: Instructors can provide class grade distributions and failure rates Focus on the present: Less emphasis to be put on the future
Course Design (41%) Changes to course design and assessment structure to support students in engaging with and bouncing back from failure.	Teaching: Teach students skills of resilience and self‐regulation, task prioritization, and the importance of failure Opportunities/options: Provide students opportunities for second changes (e.g., bonus marks, resubmissions) Assessments: Aspects related to the design of exams, tests, and assignments to best support learning from failure (e.g., practice or trial assessments as a measure of understanding and preparedness, even pacing of assessment deadlines) Lectures: Lectures that incorporate themes of failure Reflection: Students reflect on past failures through surveys or activities Marking: The provision of detailed feedback on assessments, flexibility in course policies regarding marking Weight: Incorporation of flexible grading schemes and more low‐weight assessments Tutorials: Tutorials that focus on themes of failure Transitions: Helping students transition from high school to university Office hours: Instructors to host and market office hours to assist students with course material Collaboration over competition: Foster an atmosphere where peers are not considered competitors, but collaborators Rewards: Incentivize students to think and talk about failure
Support/Resources (19%) Requests for formal and informal assistance for reviewing course material, receiving academic counseling, and building support networks.	Guidance/reassurance: Guidance that is not directly related to the course (e.g., academic advisors, student success representatives to provide advice on career pathways, upper‐year students to provide advice on past obstacles) Groups/clubs: Aspects related to peer‐to‐peer support (e.g., study sessions) Workshops: Educational sessions that teach students about stress relief and other coping mechanisms related to failure Resources supporting mental health: May include wellness booths
Change Norms (13%) Challenging stereotypes and changing the language often associated with failure	Acceptance of failure: Professors and peers accepting student failures (e.g., professors reassure students that it is okay to fail) Honest advertising: Publicize failures of students and their improvement from those failures Language: Use positive language when discussing failure Promote risk taking: Encourage students to step outside of their comfort zone at times
Expectations/Pressure (10%) Reduction of the expectations/pressures placed on students by professors, family members, or themselves	Academic pressures/expectations: Reduced emphasis on grades, instructor conversations stressing the rigor and challenging nature of university, and the expectation (whether self‐realized or due to external pressures) for students to maintain a certain GPA and full course load Peer pressure/expectations: Fear of falling behind one’s peers (whether self‐realized or due to external pressures) Self‐pressure: Pressure against oneself. Perfectionism falls in this subtheme. Career pressures/expectations: The expectation that one must have a certain level of success to obtain a career goal Familial pressures/expectations: Pressures/expectations from one’s family to succeed Societal pressure
Institutional (9%) Changes to institutional practices/policies to better support failure	University policies: Eliminate policies related to requirements of class grade distributions Academic transcript: Remove evidence of failure or only include the higher grade from a second‐course attempt when calculating one’s GPA Program design: Related to the overall program design/major (e.g., retaking a course in order to stay within a program) Program enrollment: Eliminate GPA‐specific majors/minors/specialist requirements to enroll in certain programs Financial costs: Students who feel they cannot afford to fail due to high financial costs associated with failure Probation: Reduce stigma of academic probation Exams: Eliminate all exams
Individual Responsibility (3%) When the individual is accountable for their actions and take initiative improve their circumstance	Self‐growth: Using failure as an opportunity to reflect, modify, and improve upon study habits Calmness and relaxation: Taking steps to minimize stress surrounding academic obligations
Nothing (1%) Nothing can be done to destigmatize failure	Adequate effort: All possible efforts have been made by the university. This issue must be resolved by the student population Failure negative by nature: Failure will always carry a negative connotation Grades are permanent: Marks remain on a transcript. Therefore, nothing can be done Nothing at all: Nothing can be done to destigmatize failure

^a^ Sum of response rates for each main theme does not equal 100% as some responses included multiple themes. Two students responded with ‘I don’t know’.

‘Course design’ (41%) suggested changes to course instruction that incorporate concepts of resilience and failing well, as shown by the subtheme ‘Teaching.’ ‘Support/resources’ (19%) highlighted a desire for formal and informal assistance outside the classroom such as guidance/counseling services and student groups. ‘Change norms’ (13%) suggested a use of more positive language around failure and risk taking and an increased emphasis on rewarding effort in addition to accuracy. ‘Expectations/pressure’ (10%) encompassed suggestions to reduce the pressure from professors, family members, and peers to only achieve success. One student stated, ‘To decrease the stigma of failure at university, the notion of academic competition needs to be decreased…Students are viewing their peers as competition so they do not empathize with others when they fail and simply assume that people who are failing are incapable. This couldn’t be further from the truth as people have varying circumstances which affect their academic history. Rather than promoting competition, collaboration should be emphasized in the university’.

‘Institutional’ (9%) suggestions described changes to university policies regarding course failure and program requirements. Responses related to ‘individual responsibility’ (3%) suggested it is the student/individual who should be held accountable for their actions and take initiative to resolve their own issues. Lastly, few responses (1%) suggested that nothing can be done within the context of the university to reduce the stigma of failure. Subthemes included the fact that adequate effort has already been made by the university, failure is an undesirable outcome that will always hold a negative connotation, and the fact that grades (and associated consequences of poor grades) are a permanent fixture of the university structure.

Based on responses representative of the themes of ‘institutional’ and ‘individual responsibility’, opinions on existing institutional support services were mixed. For example, one student stated, ‘I don't believe that there is much more that the university can do in order to decrease the stigma around failure. The university strongly advocates/advertises the fact that there are a multitude of resources at a student's disposal that can help them if they feel as though they are failing in their academics. It is up to the student themselves to recognize that they need assistance and make the effort to reach out’. Yet another student stated, ‘I feel that the procedures to help get students back on track or the other options are not as “surfaced” or known [as they] could be’.

### Suggestions to reduce the stigma of failure beyond the university context

Eight themes were identified for addressing stigma beyond the university context (Table 3). Similar to within the university context, ‘discussion/conversation’ (37%) was the most common theme. ‘Support’ (27%) described means of receiving assistance in coping with failure, whether that be through failure‐centered workshops, support from friends and family, or mental health resources. Suggestions representative of ‘changing norms’ (26%) advocated for a shift in societal perspectives on failure. For example, one student wrote, ‘If failure was met with less harsh judgement and punishment, and instead was regarded as an opportunity to better oneself, then it would not be seen as such a horrible thing’.

**Table 3:** Coded themes for student perspectives on decreasing stigma of failure beyond the university context (*n* = 550).

Main Theme (includes percentage of responses coded to each theme^a^)	Subthemes (in order of rank)
Discussion (37%) Inclusion of failure narratives. Increased communication on the topic of failure and resilience.	Experiences: Sharing experiences to normalize failure and connect with others Positive approach: Using positive language and viewpoints when discussing failure Open discussion: Honest conversation. Openness to listening to experiences of failure. Statistics: Showcasing failure and improvement rates
Support (27%) Resources and support networks to assist in coping with failure	Guidance/reassurance: The provision of advice or encouragement Resource/service: A system of centers, support staff, or workshops to help cope with, reflect on, or bounce back from failure Family: The ability to rely on family following failure Trials/opportunities: Opportunities beyond academia to attempt tasks and take risks without severe consequences Peers/friends: The ability to rely on peers, colleagues, and friends following failure Groups: A safe space or gathering for people to connect and discuss failure Mental health: Increase awareness and support for mental health
Changing Norms (26%) Advocate for change regarding the existing negative stigma of failure	Understanding/acceptance: Normalize compassion for those who fail Shifting emphasis away from grades: Minimize preoccupation with grades, and instead stress the value of happiness, experience, and creative intelligence Language: Use more positive language in discussions of failure Knowledge accuracy: Have educational systems place greater value on understanding and growth over accuracy and correctness Reward effort: Appreciation of effort and participation. Celebration of failures that have led to successes. Reduce consequences: Where possible, reduce the cost or negative effects of failure Promote risk taking: Encouragement to step outside of one’s comfort zone at times
Education (21%) Initiatives to reduce the stigma of failure through research, or the incorporation of strategies to develop resilience in the educational system (e.g., primary and secondary school curricula)	Resilience: Learned skills to recover or bounce back from failure Importance of failure: Emphasis on how failure is part of the learning process Start young: Introducing topics of failure at younger grades Curriculum/course design: Implementing opportunity for failure within school curricula Research: Research programs focused on the importance of failure and efficacy of failure interventions Recognizing patterns: Strategies to learn how to avoid repeating the same mistakes/experiencing the same failures without experiencing growth
Media (20%) Incorporation of failure narratives in popular culture, social media, and advertisements	Important figures: Sharing of failure experiences by celebrities and influential people Pop culture: Communicating the importance of failure through shows, movies, music, etc. Advertising: Posters, campaigns, ads, etc. Platforms: Use of various platforms like social media, public speeches, and conferences
Expectation/Pressures (10%) Reduction of external expectations/pressures to succeed	Societal: Reduce negative connotation often associated with failure, societal expectations (e.g., career, salary, status) Family: Parents, siblings, relatives Career: Colleagues, employers Self: Personal goals/standards, perfectionism Academic: Reduce comparisons of achievements (grades, GPA) among students Friends: Eliminate failure judgment from peers/friends
Individual Responsibility (7%) When the individual is accountable for their actions and take initiative improve their circumstance	Self‐perception: Confidence and self‐motivation. Avoid comparing oneself to others. Work habits: Preparedness, reflection, and revision of study habits Lifestyle: Exercise, sleep, meditation, etc.
Nothing (1%) Nothing can be done to destigmatize failure	[No subthemes identified]

^a^ Sum of response rates for each main theme does not equal 100% as some responses included multiple themes. Five students responded with ‘I don’t know’.

The theme of ‘education’ (21%) included suggestions to teach the value of failure and resilience in contexts other than the university classroom. One student suggested ‘…at a young age, children should be taught in schools that it is okay to fail’. The theme of ‘media’ (20%) included suggestions of how to connect and promote failing well using public forums. Subthemes included the use of celebrities, pop culture media, advertising, and social media platforms to promote the value of failure.

A reduction in ‘expectations/pressure’ was described in 10% of responses, and ‘individual responsibility’ represented 7% of responses. No subthemes were identified in the least common theme, ‘nothing’ (1%); students described various reasons as to why they felt nothing could be done to reduce the stigma of failure, including the idea that a person’s value is tied to their success and failure is negative by nature.

### Comparison between the university and nonuniversity contexts

**Fig. 2:**
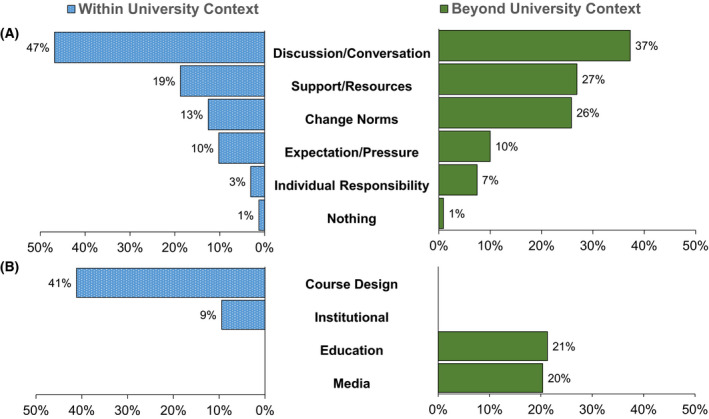
Alignment of student perspectives on how to reduce the stigma of failure within and outside the university context (*n* = 550). Ten themes were identified in students’ responses of which eight were common to both contexts (A) and four were unique to a single context (B). Responses that described multiple distinct themes were counted multiple times, leading to total percentages for each context that exceed 100%.

A comparison of themes between the university context and non‐university context revealed significant alignment: eight themes were common to both contexts, two were unique to the within university context, and two were unique to the beyond university context (Figure 2). The eight themes shared between the two contexts held the same rank order.

### Resources to help students embrace and bounce back from failure

In student suggestions for specific resources to help embrace failure, the most common theme was a need for a strong support network (45%) through peers, instructors, and family (Table S1). Students also suggest incorporating failure narratives (15%) through sharing of personal stories or those of public figures, providing organized events such as study groups and failure‐based workshops (13%), and increasing accessibility to mental health resources (10%). An additional 12 suggestions were identified in student responses, although they were represented in fewer than 10% of responses (Table S1).

## Discussion

Experiencing failure is an important part of the learning process, and core to the ability to develop as a successful scientist [1, 2]. If we are to train undergraduate students to be scientists, we need to teach them to interact with failure as a productive part of the scientific process. Despite the potential benefits of failure, many undergraduate students fear it [5, 24], thus making it challenging for instructors to teach their students to learn from and embrace failure. This fear may be driven by the negative stigma associated with failure, as well as the internal and external pressures students face to succeed. We found that societal and familial pressures were the greatest contributing external factors to students’ fear of failure. Student perspectives on reducing the stigma of failure were united in major themes of normalizing failure as part of the learning process, reducing consequences of failure both within and beyond the university context, and reducing competition among peers.

### Factors contributing to students' fear of failure

As a result of societal and familial pressures to succeed, students fear possible repercussions of being seen in a negative light by those around them. Many students indicated they are not comfortable with failure based on their perception that society does not value failure as much as success. Experiences with failure can provide valuable information and learning opportunities, yet individuals often share stories of successes and withhold information on failures [25, 26, 27]. For many, feelings of shame and embarrassment following failure is rooted in fear of upsetting or becoming distanced from individuals that are most important to them [28]. This fear may be heightened in students who tend toward perfectionism as a constant concern for high achievement can lead to risk avoidance, self‐doubt, and fear of failing to meet expectations [29, 30]. Similarly, students who experience low self‐esteem may be more likely to believe that sharing experiences of failure will negatively impact their interpersonal relationships as others will value them less following their admission of failure [31].

Fear of failure associated with reputational costs, being judged or ridiculed, or disappointing others is exacerbated in an unsupportive peer environment. In more competitive environments, individuals are particularly unlikely to share experiences of failure or poor performance [27]. In addition to peer‐to‐peer interactions, fear of failure can cause students to be hesitant in discussing failures with parental figures [25]. Although our Likert questions asked generally of the role of family in contributing to one’s fear of failure, most accompanying open‐ended responses described parent–student relationships specifically. The shame and fear of failure, driven by societal stigma and the expectations of self and others, are key motivators in the hesitancy to disclose personal experiences with failure [25].

### The privilege of being able to fail

Although failure is beneficial for learning and foundational to the scientific process [1, 8], instructors must be mindful of the fact that consequences of failure are not equal for everyone. One of the main challenges for instructors in reducing students’ fear of failure is the fact that students are in an institutional structure for which grades remain the primary proof of learning. Furthermore, students who come from a more financially privileged background may have opportunities to retake courses, an option not feasible for some of their peers. Additionally, Kim and Sax [32] found that compared to students from lower‐ or middle‐class families, students from upper‐class families and/or those with parents who were college or university‐educated were more likely to engage with their professors to ask for help and receive additional support when facing challenges. In addition to socioeconomic status, fear of failure may disproportionately affect women, ethnic minorities, and other marginalized populations [2, 16, 17]. This is of particular concern as these groups are already underrepresented in the sciences, and a fear of failure may reduce their likelihood of pursuing a STEM degree [16, 17, 33, 34]. Since a student’s socioeconomic status, culture, and gender can influence their ability to cope with and bounce back from failure, to better support all students, instructors could strive to foster an inclusive classroom environment supportive of marginalized students who may otherwise be hesitant to seek help.

### Reducing the stigma of failure within the classroom

Our data suggest that societal roles and familial pressure weigh heavily on students’ fear of failure. However, this does not mean that instructors hold little responsibility in reducing the negative stigma of failure. Implementing effective classroom strategies may create opportunity to decrease the stigma of failure while modeling meaningful and impactful reflective learning strategies.

Both within and beyond the university contexts, the most common student suggestion to reduce the stigma of failure was increased communication. Similarly, in the open‐ended question on resources to help students embrace and bounce back from failure, many student responses cited the need to increase failure narratives in the classroom. Common suggestions were for professors or upper‐year students to share their failures with introductory‐level students, reinforcing that one is not alone in facing academic and personal challenges. Instructors may also stress the importance of failure in the learning process by providing examples of failures in the sciences and emphasizing their value to the process of discovery, thus reinforcing the role of failure as part of progression and growth.

Although this research is designed to inspire the development of interventions, we can draw upon some existing pedagogical interventions that are relevant and applicable to fear of failure. Failure interventions can be used to reduce the negative emotions students may experience before attempting a challenge [35]. For instance, assessments designed for productive failure can help students embrace failure, improve their understanding of course material, identify issues with study habits, and encourage them to seek support [8, 9, 12]. Specifically, low‐weight and scaffolded formative assessments can allow students to experience failure in a low‐risk scenario, providing opportunity for regular reflection and supporting the development of resilience [36]. This strategy aligns with student suggestions to reduce the stigma of failure within the university context through course design modification: reducing the reliance on high‐weight summative assessments, increasing the overall number of assessments over the term, and providing more opportunities for failure and improvement in a low‐risk environment. To encourage the development of resilience, instructors can also offer opportunities for students to engage in higher order thinking challenges that are coupled with student choice or grading flexibility [37]. Such an approach creates a more manageable level of challenge for students [38]. Finally, encouraging metacognitive processing can help students learn from failures and modify their study habits to optimize learning. For example, instructors may offer questionnaires for students to reflect on test performance, critically analyze the way they prepared for the test, and suggest ways they may alter their study strategies in preparation for upcoming evaluations. Such reflection can help students evaluate past failures and help develop skills of self‐regulation [36].

### Building community to reduce the stigma of failure

Beyond creating space for the sharing of failure experiences between instructors and students, one can foster a sense of community in the classroom to encourage peer‐to‐peer communication and support. Students tend to experience reduced anxiety and fear of failure in a collaborative versus competitive learning environment [35, 39, 40]. A classroom environment built on kindness and compassion is most productive for the development of resilience [39]. To create an inclusive learning environment that reduces the stigma of failure, instructors can incorporate greater flexibility in their course policies. Greater leeway in policies, such as extensions and late penalties, can help accommodate students who may be struggling, particularly those who may not vocalize their need for help as a consequence of differences in power and privilege.

Inequities in inclusion are long‐standing issues for equity‐seeking groups in post‐secondary education. One can create a more inclusive environment by minimizing threats in the classroom, sharing experiences, celebrating differences, and making personal contact with students [39]. Community building can also address barriers that would otherwise impact the experience a student has with their instructor, including imbalances in accessing help [32]. Although studied in the context of social media and mental health, Naslund et al. [41] found that peer‐to‐peer support helped to reduce stigma associated with mental illness, increased one’s sense of belonging, and led to the sharing of coping strategies with others. These principles may also apply to the development of peer support in reducing the stigma of failure.

### Reducing the stigma of failure beyond the classroom

Institutional‐level changes were frequently described in student suggestions to reduce the stigma of failure within the university context. However, opinions on the efficacy and accessibility of existing institutional support services were mixed. This suggests inconsistencies in student engagement or familiarity with these resources. Institutions may consider implementing new methods of increasing awareness of services with the specific goal of targeting student populations that are traditionally underrepresented in help seeking. Further, instructors can familiarize themselves with campus resources such as counseling, academic advising, and relevant workshops, to share with one’s students [42].

A unique theme identified in student responses for beyond the university context was that of media. Many students suggested using social media as a tool to increase awareness of the stigma of failure and to use this as a platform to share experiences of failure beyond the classroom. Previous research has found the use of social media an effective tool in garnering awareness, promoting conversation, and encouraging help seeking related to mental health concerns [43]. A similar approach may be used to normalize failure, help seeking, and risk taking in undergraduate student populations. Instructors may also consider using or promoting social media tools as a means to continue peer‐to‐peer communication beyond the classroom.

Failure is a key aspect of the scientific method that should be viewed as part of the learning process rather than something to be feared or avoided. By drawing upon the literature on the learning process, science educators can find inspiration for how to better support students in learning how to use failure to improve their skills as scientists and as a core part of the reflective learning process. Teaching undergraduate science students to learn from and embrace failure is a challenging task, but it remains necessary to foster the growth of successful scientists. Student perspectives of how to decrease the stigma of failure within and outside the university contexts highlight several aspects of student life wherein room exists to implement changes, including promoting discussion, providing more effective sources of support, and fostering community‐based classroom environments. It is important to recognize that factors beyond the classroom are often associated with student fears, but instructors can help mitigate the negative stigma of failure by fostering a supportive and inclusive student‐centered learning environment that encourages collaboration, discussion, and normalization of failure narratives, both within science and beyond.

## Conflict of interest

The authors declare no conflict of interest.

## Author contributions

NL and FR defined the research objective and designed the survey. FR performed the data collection. KN, SD, RP, MM, and ZM completed the data analyses. SD and RP wrote a first draft of the manuscript; and KN, NL, and FR wrote the final draft of the manuscript.

## Supporting information


**Table S1.** Coded themes for student responses to the open‐ended question, ‘What resources do you think students need to help them embrace and bounce back from failure?’ (*n* = 880).

## Data Availability

The data that support the findings of this study are openly available in figshare at http://doi.org/10.6084/m9.figshare.17118917
