# Supplementary material for: Science students' perspectives on how to decrease the stigma of failure
Source: FEBS Open Bio. 2021 Dec 13;12(1):24–37. doi: 10.1002/2211-5463.13345 (PMC8727946; doi:10.1002/2211-5463.13345)
Supplement: Supplementary file 1 — Table S1. Coded themes for student responses to the open‐ended question, ‘What resources do you think students need to help them embrace and bounce back from failure?’ (n = 880). [file FEB4-12-24-s001.docx]

**Supplementary Material**

Science students’ perspectives on how to decrease the stigma of failure

Krystal Nunes^1*^, Sherry Du^1^, Riya Philip^1^, Mohammed Majd Mourad^1^, Zainab Mansoor^1^, Nicole Laliberté^1^, Fiona Rawle^1^

^1^Department of Biology
University of Toronto Mississauga

3359 Mississauga Road

Mississauga, ON

L5L 1C6 Canada

* indicates corresponding author

**Table S1.** Coded themes for student responses to the open-ended question, “What resources do you think students need to help them embrace and bounce back from failure?” (n = 880).

| **Theme** (includes percentage of responses coded to each theme*) | **Description of Theme** | **Example Response** |
| --- | --- | --- |
| **Support network (45.1%)** | Encouragement from peers, instructors, teaching assistants, parents, etc. | “Support! Personally speaking, nothing rejuvenates me more than having a family member or friend tell me that I'm doing just fine and that I should definitely keep going in whatever I'm doing because I'm on track to succeed. Support systems are crucial” |
| **Failure narratives (15.0%)** | Sharing past failures of professors and/or upper-year students. Incorporate stories of failures experienced by famous scientists or public figures into course content | “Professors and upper-year students sharing their stories and perspective on life and how they became who they are. Everyone will have some kind of failure and those stories will motivate students” |
| **Study groups/workshops (12.5%)** | Organized events focused on course content review and/or strategies for studying, self-regulation, and resilience. | “I feel as though we could use some form of guidance. Something like facilitated study groups, pear academic leaders, and other student resources specifically for giving advice and helping students lay out a path and plan of action for their work” |
| **Mental health resources (10.1%)** | Access to counsellors/therapists. Events to help students reduce stress and anxiety (e.g., yoga, mediation) | “I think when dealing with failure, many students are struggling with their mental health. If they can have someone like a counselor that can listen to their problems, understand their mental state and give them motivating advice, I think that will help them” |
| **Course flexibility (8.9%)** | Opportunities to improve scores/past performance through flexible grading schemes (e.g., dropping lowest mark), or assessments that allow for improvement (e.g., option to revise and resubmit). | “I think students need safe places to fail academically. By offering opportunities to redo assignments and extra academic support, students will feel less overwhelmed by the prospect of failure, allowing them to embrace the education that comes with failure” |
| **Academic counselling (7.5%)** | Access to staff that can provide advice/guidance for students following a failure. | “Specific counsellors that can help students with their next steps after a failure, to help them figure out what to do, how to do it, and also help them realize that it is not the end of the world.” |
| **Strategies to develop resilience (4.7%)** | Concrete suggestions of how students can bounce back from failure and work to develop skills of self-regulation and resilience. | “I think students need real workshops and lessons on how to embrace and bounce back from failure. What does it look like? What is the dialogue I should have in my head after failing? What will it feel like? What actions should I be doing?” |
| **Increased course feedback (4.7%)** | Greater detail in marking rubrics and comments provided on assessments. Access to instructors/TAs to discuss areas for improvement. | “More comments and constructive criticism are the most useful tool to correcting one's failure because often we don't know what we did wrong to fail in the first place. It takes someone with an outside point of view to show us what we did wrong.” |
| **Reflections/productive failure assessments (3.0%)** | Classroom interventions designed to increase resilience. This may include productive failure assessments (those designed to be challenging to have students engage with failure) or reflective assessments designed to engage metacognition. | “Students need more curriculars that accept failure. For example, activities that are set up for you to fail, so that you can learn from them in the end.” |
| **Test/assessment prep materials (2.2%)** | Recommendations for more detailed assignment instructions, practice tests, and access to past student submissions showing the process of working through solutions. | “I think that students need to understand the grading scheme better while knowing how to answer questions effectively. A beneficial source will have to be old tests and quizzes from other people and [not just a] perfect answer provided by graders” |
| **Time to process (1.5%)** | Providing breaks or slower pacing of course material following a substantial assessment | “By providing them with time enough to focus on what they failed at. University is so fast paced, we barely have time to rest, but rather after one assignment we quickly jump onto the next. If we fail at something we don't have time enough to fully ponder” |
| **Awareness of existing services (1.3%)** | The belief that resources to assist students in embracing failure already exist, but they need to be promoted. | “I think the school already offers plenty of services to help students rebound from failure, it’s just a matter of making sure students actually know these services exist.” |
| **Scaffolded/low-weight assessments (1.0%)** | Opportunities for students to experience low-risk failures. This includes scaffolded assessments consisting of smaller tasks with regular feedback. | “A major resource would be mock assessments or step-by-step assignments that both involve a small portion of their grade and work more to set a current level knowledge. In both cases, rather than a rubric focusing on marks, comments on areas of improvement” |
| **Nothing (0.6%)** | No resources exist that will help students embrace and bounce back from failure. | “I feel that more so than resources, one must be able to understand their situation from a third person point of view. They must be able to accept that they failed, and overcome it. In terms of resources, realistically, I don't think there are any” |
| **Financial support (0.5%)** | Minimize concerns of the monetary repercussions of failure, such as the cost of repeating a post-secondary course. | “Focusing mainly on school I believe more of a safety net would be good for students. If we didn't have to worry about money as a consequence we would be less stressed and worried about messing up in schooling. In addition to that, with less money concerns” |
| **Data sharing (0.3%)** | Providing students with information on grade distributions. | “Comprehensive information about their failure so they can see where they went wrong as well as data about other people’s failures so they know they weren’t alone” |
| **Nondescript (3%)** | Responses that did not answer the question. | N/A |

^*Sum of response rates for each main theme do not equal 100% as some responses included multiple themes.^
